# Pomegranate Mesocarp against Colitis-Induced Visceral Pain in Rats: Effects of a Decoction and Its Fractions

**DOI:** 10.3390/ijms21124304

**Published:** 2020-06-17

**Authors:** Carmen Parisio, Elena Lucarini, Laura Micheli, Alessandra Toti, Mohamad Khatib, Nadia Mulinacci, Laura Calosi, Daniele Bani, Lorenzo Di Cesare Mannelli, Carla Ghelardini

**Affiliations:** 1 Department of Neuroscience, Psychology, Drug Research and Child Health-NEUROFARBA-Pharmacology and Toxicology Section, University of Florence, Viale Pieraccini 6, 50139 Florence, Italy; carmen.parisio@unifi.it (C.P.); elena.lucarini@unifi.it (E.L.); laura.micheli@unifi.it (L.M.); alessandra.toti@unifi.it (A.T.); carla.ghelardini@unifi.it (C.G.); 2 Department of Neuroscience, Psychology, Drug Research and Child Health-NEUROFARBA-Pharmaceutical and Nutraceutical Division, University of Florence, Via Ugo Schiff 6, 50019 Florence, Italy; mohammadtttt@yahoo.com (M.K.); nadia.mulinacci@unifi.it (N.M.); 3 Department of Experimental & Clinical Medicine, Section of Anatomy & Histology & Research Unit of Histology & Embryology, University of Florence, Viale Pieraccini 6, 50139 Florence, Italy; laura.calosi@unifi.it (L.C.); daniele.bani@unifi.it (D.B.)

**Keywords:** polysaccharides, ellagitannins, punicalagin, chronic visceral pain, colitis, DNBS, IBDs, IBS, rats

## Abstract

The management of chronic visceral pain related to Inflammatory Bowel Diseases or Irritable Bowel Syndrome is still a clinical problem and new therapeutic strategies continue to be investigated. In the present study, the efficacy of a pomegranate decoction and of its polysaccharide and ellagitannin components in preventing the development of colitis-induced abdominal pain in rats was evaluated. After colitis induction by 2,4-dinitrobenzenesulfonic acid (DNBS), the pomegranate decoction (300 mg kg^−1^), polysaccharides (300 mg kg^−1^), and ellagitannins (45 mg kg^−1^) were orally administered for 14 days. Repeated treatment with decoction reduced visceral hypersensitivity in the colitic animals both at 7 and 14 days. Similar efficacy was shown by polysaccharides, but with lower potency. Ellagitannins administered at dose equivalent to decoction content showed higher efficacy in reducing the development of visceral pain. Macroscopic and microscopic evaluations performed on the colon 14 days after the damage showed that all three preparations reduced the overall amount of mast cells, the number of degranulated mast cells, and the density of collagen fibers in the mucosal stroma. Although ellagitannins seem to be responsible for most of the beneficial effects of pomegranate on DNBS-induced colitis, the polysaccharides support and enhance its effect. Therefore, pomegranate mesocarp preparations could represent a complementary approach to conventional therapies for promoting abdominal pain relief.

## 1. Introduction

Pomegranate is a shrub belonging to the Punicaceae family, that includes *Punica granatum* (edible pomegranate), which is indigenous to Iran and Mediterranean regions, and *Punica protopunica* (inedible), cultivated in Socotra islands in Pacific Ocean [1]. The pomegranate tree and its fruit have been extensively used as a source of traditional medicine [2,3]; today, scientific results strongly support its medicinal relevance. Among the pomegranate chemical components, ellagic acid, ellagitannins, punicic acid, anthocyanins, flavonols, flavan-3-ols, and flavones seem to be the ones responsible for most of its therapeutic benefits [4,5]. These products were found in all fruit parts, including the fruit peel (ellagitannis, flavonoids, anthocyanins), seeds (fatty acids, lipids), and membranous walls (mostly ellagitannins) [6]. Ellagitannins, which include the minor tannins called punicalin and gallagic acid, have the ability to be hydrolyzed to ellagic acid at the intestinal level, resulting in a prolonged release of this acid into the blood [7,8]. Ellagic acid, metabolized in vivo by the gut microbiota to yield urolithins, also shows dose-related antinociceptive actions with both peripheral and central components [9]. Gainok et al. [10] described the antinociceptive effects of ellagic acid in the rat hot-plate model. Moreover, ellagic acid was also able to significantly decrease the abdominal irritation induced by acetic acid in mice [11]. Pomegranate possesses antioxidant [12,13], antimicrobial, and prebiotic effects [14], as well as spasmogenic and spasmolytic activities [15]. Recently, the potential beneficial properties of pomegranate in several gastrointestinal diseases have become increasingly interesting.

Visceral abdominal hypersensitivity is a main symptom of gastrointestinal diseases, such as Inflammatory Bowel Diseases (IBDs) and Irritable Bowel Syndrome (IBS), chronic conditions with increasing incidence, prevalence, and severity in recent years [16,17] particularly in Canada, Australia, and Europe [18]. While IBDs are heterogeneous chronic inflammatory disorders of the gastrointestinal tract, involving genetic, environmental, and immunological alterations [19,20], IBS is a functional disease related to immune activation and defective barrier function apparently without structural abnormalities [21,22]. As chronic conditions affecting the gut, IBDs and IBS present some clinical overlap like abdominal pain, alterations of intestinal transit, and they both tend to be diagnosed in young people [23,24]. In IBDs, patients upregulated expression of cytokines (such as TNF-α, IL-6, IL-12, IL-17) and substance P [25,26], and alterations in gut microbiota [27] were observed, as well as significantly increased numbers of mast cells in the mucosa of the ileum and colon [28], even in IBS patients [29]. Mast cells play a very important role in the regulation of intestinal permeability and, being below the intestinal mucosa barrier, can be activated by microbial antigens [30,31]. In addition, levels of tight junction proteins are significantly downregulated in IBDs patients, leading to increased gut permeability to microbial ligands and noxious metabolites, resulting in systemic inflammatory responses [32,33]. Although the knowledge of these pathologies is progressing, the management of visceral pain in patients is still a clinical problem today [34].

Hypothesizing a possible effect of pomegranate products against visceral pain, the purpose of this study was to evaluate the efficacy of a dry whole pomegranate decoction (obtained from mesocarp of the Wonderful variety) and of its main components, polysaccharides and ellagitannins, in preventing the development of abdominal pain induced in rats by the intracolonic instillation of 2,4-dinitrobenzenesulfonic acid (DNBS) [35].

## 2. Results

### 2.1. Composition of the Ellagitannins in Dry Decoction and in the Ellagitannin Fraction

**Figure 1:**
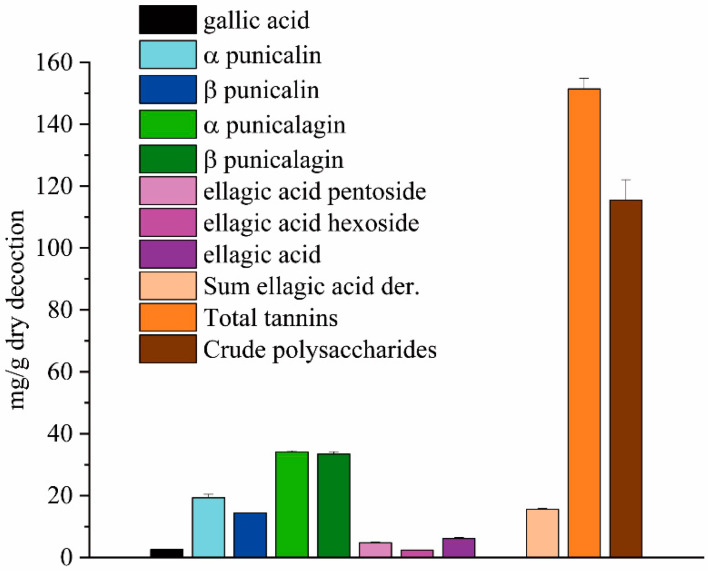
Composition of the dry decoction in terms of phenolic constituents and crude polysaccharides. Distribution of the phenolic compounds, total phenolic content, and crude polysaccharide amount. The data are expressed as a mean of a triplicate, as mg/g dry decoction.

The samples tested in the animal models were chosen to evaluate the effect of the decoction, which was considered as phytocomplex, and to verify the role of its two main bioactive fractions: the ellagitannins and polysaccharides. Figure 1 summarizes the composition of the dry decoction in terms of its phenolic constituents and crude polysaccharides. Regarding the phenolic fraction, the sum of a + b punicalagin represents over 60% of the phenols present in the decoction. These ellagitannins typical of pomegranate have little presence in the arils used to produce the juice, but are mainly concentrated in the mesocarp of the fruit.

### 2.2. Effect of Repeated Treatment with Pomegranate-Based Preparations on Visceral Hypersensitivity

**Figure 2:**
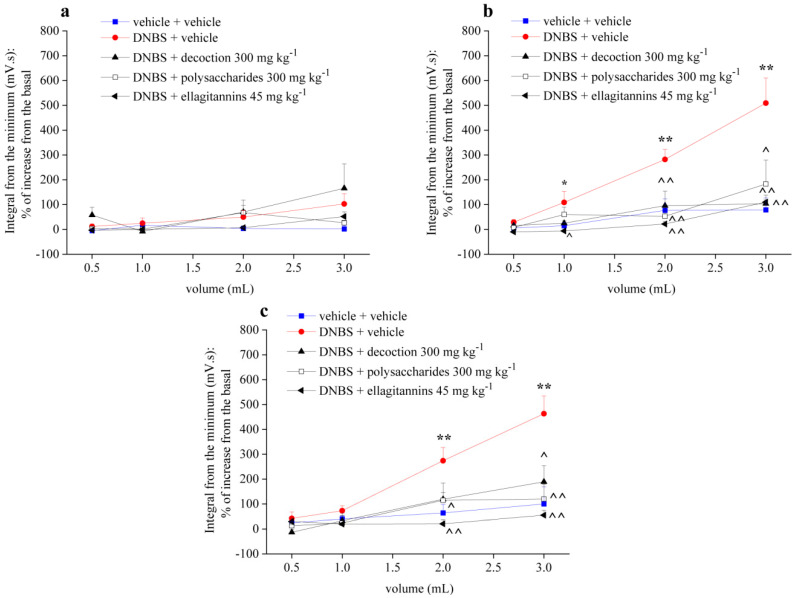
Effect of repeated treatment with pomegranate-based preparations on visceral hypersensitivity. Tests were performed before the treatments (**a**), 7 (**b**) and 14 (**c**) days after the damage induction, measuring the Visceromotor Response (VMR) to the Colorectal Balloon Distension (CRD). Each value is the mean ± S.E.M. and represents the mean of six rats per group. * *p* < 0.05 and ** *p* < 0.01 vs. vehicle + vehicle treated animals. ^ *p* < 0.05 and ^^ *p* < 0.01 vs. 2,4-dinitrobenzenesulfonic acid (DNBS) + vehicle treated animals.

Figure 2 shows the effect of repeated administration of pomegranate whole decoction (300 mg kg^−1^) and its derived polysaccharides (300 mg kg^−1^) and ellagitannins (45 mg kg^−1^) fractions on visceral hypersensitivity induced by intrarectal administration of DNBS (30 mg dissolved in 0.25 mL EtOH 50%) in the rats. Pomegranate preparations were orally administered once a day for 14 days, starting from DNBS injection. Visceral sensitivity was assessed by measuring the VMR to CRD before DNBS injection (Figure 2a) as well as 7 (Figure 2b) and 14 (Figure 2c) days after.

As shown in the pretest, the experimental groups had the same sensitive threshold before the treatments (Figure 2a). Control animals were almost insensitive to the stimuli (Figure 2), an indication that the increase in visceromotor sensitivity in other experimental groups was not caused by the method procedures. In contrast to control group, DNBS-treated animals showed a strong abdominal contraction in response to the colorectal distension both 7 (Figure 2b) and 14 (Figure 2c) days after DNBS injection. In animals treated with DNBS + pomegranate whole decoction 7 days after the damage (Figure 2b), the abdominal visceromotor response to colorectal distension was significantly reduced at 2 mL and 3 mL balloon inflation; on the 14th day after damage induction (Figure 2c), repeated treatment with pomegranate decoction led to significant reduction of visceral sensitivity, with 3 mL distension volume. Repeated administration with the polysaccharide fraction reduced visceral sensitivity in animals with 2 and 3 mL balloon inflation both 7 (Figure 2b) and 14 (Figure 2c) days after induction of damage, with similar efficacy to the whole decoction. In animals treated with DNBS + ellagitannin fraction, the abdominal visceromotor response to colorectal distension was considerably reduced by all distension volumes used, both 7 (Figure 2b) and 14 (Figure 2c) days after the DNBS administration.

### 2.3. Effect of Repeated Treatment with Pomegranate-Based Preparations on Abdominal Withdrawal Response

**Figure 3:**
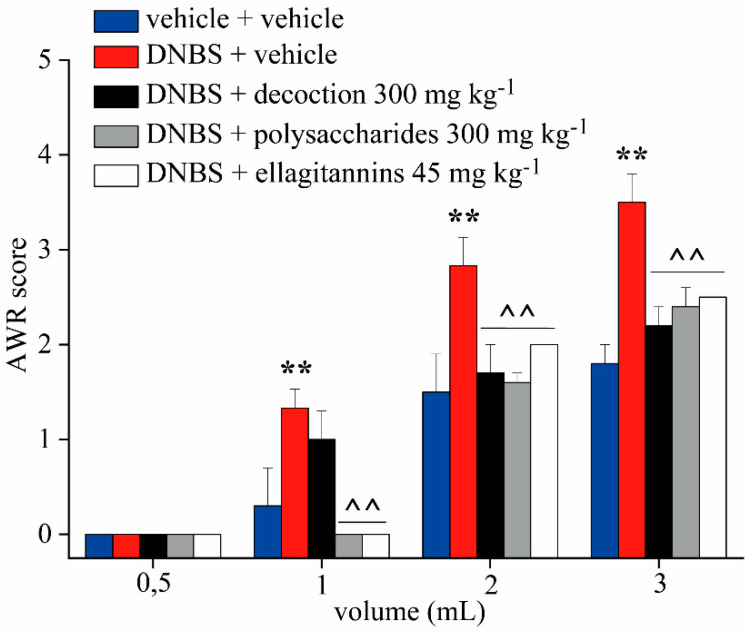
Effect of repeated treatment with pomegranate-based preparations on behavioral alterations related to pain perception. Behavioral responses to CRD were assessed Abdominal Withdrawal Reflex (AWR) measurement, using a semiquantitative score in conscious animals. Tests were performed 14 days after DNBS administration in awake animals. Each value is the mean ± S.E.M. and represents the mean of six rats per group. ** *p* < 0.01 vs. vehicle + vehicle treated animals ^^ *p* < 0.01 vs. DNBS + vehicle treated animals.

Figure 3 shows behavioral responses to graded colorectal distension (0.5, 1, 2, 3 mL distension volume) assessed via Abdominal Withdrawal Reflex (AWR, an involuntary motor reflex similar to the visceromotor reflex) using a semiquantitative score increasing with pain-related behavior. The test was performed 14 days after DNBS (30 mg in 0.25 mL EtOH 50%) administration in awake animals.

The animals treated with DNBS + vehicle showed a significantly higher AWR score at all distension volumes than the control animals. Repeated treatment with pomegranate whole decoction reduced the AWR score at 2 and 3 mL of distension volume. On the contrary, repeated treatment with two derived polysaccharides and ellagitannin fractions reduced the AWR score at all distention volumes, without showing significant difference of efficacy among them.

### 2.4. Effect of Repeated Treatment with Pomegranate-Based Preparations on Colon Damage

**Figure 4:**
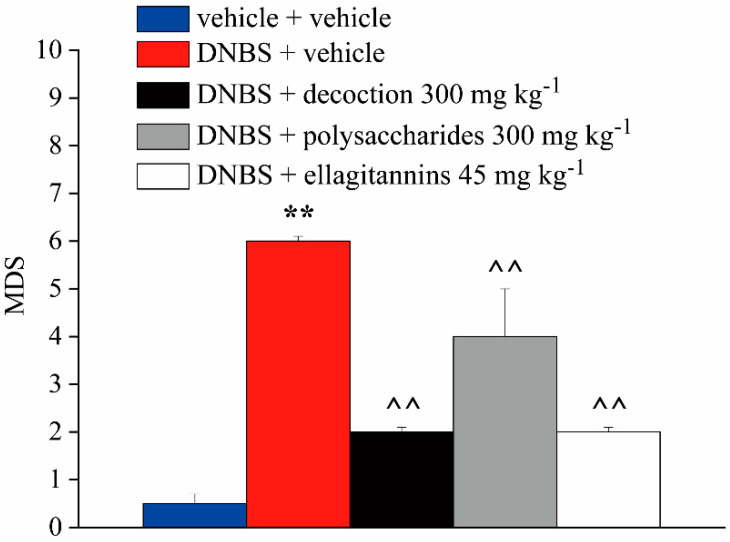
Effect of repeated treatment with pomegranate-based preparations on colon macroscopic damage. Animals were sacrificed 14 days after DNBS injection, and the colon portion was removed to perform the macroscopic analysis. The Macroscopic Damage Score (MDS) was used to quantify the tissue damage degree. Each value is the mean ± S.E.M. and represents the mean of six rats per group. ** *p* < 0.01 vs. vehicle + vehicle treated animals. ^^ *p* < 0.01 vs. DNBS + vehicle treated animals.

**Figure 5:**
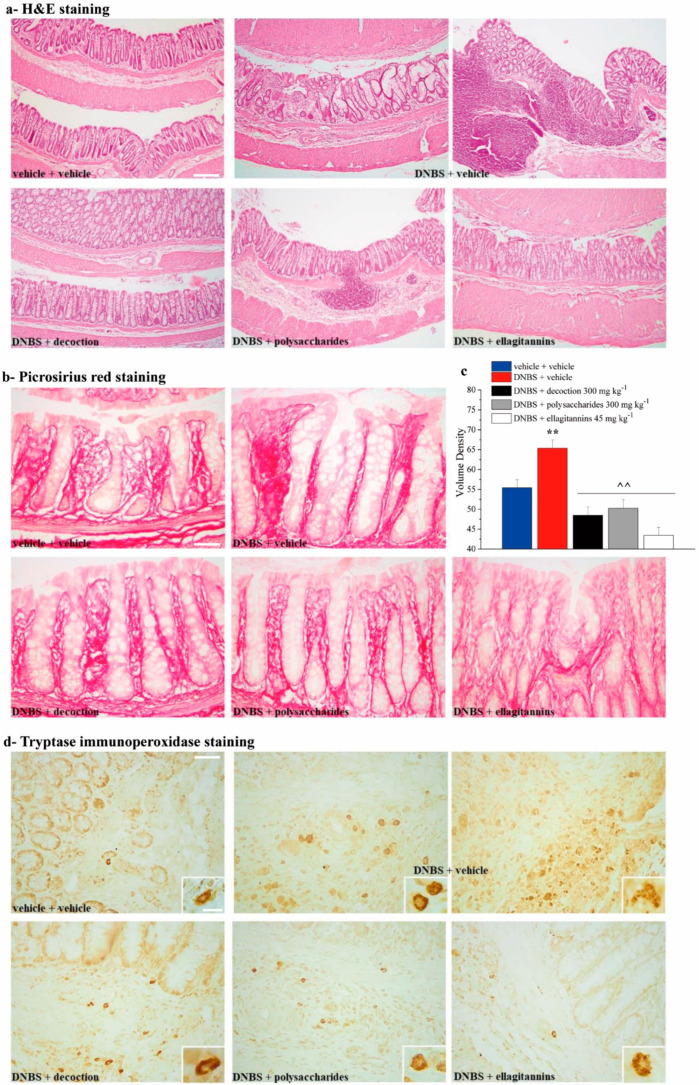
Effect of repeated treatment with pomegranate-based preparations on colon microscopic damage. Microscopic evaluations were carried out 14 days after DNBS injection by light microscopy on sections of colon. Histological evaluations were performed on sections stained with hematoxylin and eosin (**a**, scale bar: 20×), picrosirius red staining (**b**, scale bar: 40×), and tryptase immunohistochemistry (**d**, scale bar: 40×, insert: 100×). For picrosirius red analysis, a morphometric quantitative evaluation of the staining intensity (expressed as volume density) was performed on the digital images (**c**). The reported values are the means ± SEM of the measurements of individual animals (at least five images each) from the different experimental groups. ** *p* ˂ 0.01 vs. vehicle + vehicle group. ^^ *p* ˂ 0.01 vs. DNBS + vehicle group.

Figure 4 and Figure 5 show the effect of repeated administration of pomegranate whole decoction (300 mg kg^−1^) and its derived polysaccharide (300 mg kg^−1^) and ellagitannin (45 mg kg^−1^) fractions on tissue damage induced by DNBS at the colorectal level. The animals were sacrificed 14 days after DNBS injection, and the colon was harvested and processed for both macroscopic (Figure 4) and microscopic (Figure 5) histological analysis. The Macroscopic Damage Score (MDS) was used to quantify the tissue damage degree.

On the fourteenth day after the induction of the damage, the DMS of DNBS + vehicle group was significantly higher, by about six times, than the control animals score (Figure 4). In animals treated with DNBS + polysaccharide fraction, the MDS was reduced by about two times compared to that of DNBS + vehicle animals (Figure 4). Repeated treatment with both pomegranate whole decoction and its ellagitannin fraction resulted in a statistically significant reduction of MDS of about four times compared to that of DNBS + vehicle animals, without showing difference of efficacy among them (Figure 4).

Histological evaluation of the damage at the microscopic level was subsequently performed by light microscopy on sections of colon (5 μm) stained with hematoxylin and eosin, picrosirius red (PR), and tryptase immunoperoxidase, paying particular attention to intestinal inflammation, fibrosis, and increase in mast cells induced by DNBS injection.

Examination of hematoxylin and eosin stained histological sections of rat colonic samples under the different treatments (Figure 5a) showed that in control animals no reveal significant alterations at the tissue level were reveal, and this allowed to exclude that the lesions observed in the other experimental groups were consequent to the method used. On the contrary, compared with the normal features of the untreated controls, the animals treated with DNBS + vehicle showed a marked hypertrophy of mucous glands, hyperplasia of the crypts (with irregular structure and variable diameter), diffuse inflammatory infiltrate in the mucosal stroma with a large increase in neutrophils both in the epithelial cells and in the lumen of the crypts. Cotreatment with either ellagitannins, whole decoction, or polysaccharides attenuated all these histological abnormalities.

In the sections stained with the PR method for collagen fibers (Figure 5b), the specimens from the rats treated with DNBS + vehicle showed a denser collagen framework in the mucosal stroma as compared with the untreated controls, suggestive of the occurrence of fibrosis. This data was also highlighted by the morphometric analysis, that showed a significant increase in the volume density of collagen fibers in animals treated with DNBS + vehicle compared to the control group (Figure 5c). Cotreatment with either ellagitannins, whole decoction, or polysaccharides reduced the collagen fiber density.

In the sections immunostained to detect mast cells by immunoreactivity of tryptase, a specific enzyme contained in their secretion granules (Figure 5d), the specimens from the animals treated with DNBS + vehicle showed higher amounts of mast cells in the mucosal stroma than in the control counterparts, some of which featuring degranulated cells. Cotreatment with either ellagitannins, whole decoction, or polysaccharides reduced the overall amount of mast cells, which showed a substantially normal complement of tryptase-positive secretion granules.

## 3. Discussion

The present research shows the efficacy of pomegranate whole dry decoction as well as of its ellagitannin and polysaccharide fractions against colitis-induced visceral hypersensitivity in rats. The higher potency of the ellagitannin fraction in comparison to the polysaccharide fraction was highlighted. Furthermore, the repeated treatment with either whole decoction, polysaccharides, or ellagitannins significantly reduced the macroscopic colon alterations. The inflammatory infiltrate, the number, and distribution of the crypts were normalized, the volume density of collagen fibers was reduced, and the overall number of mast cells in the mucosal stroma was decreased.

The DNBS injection induces local inflammation that peaks between 3 and 7 days after the injection [36]; its intracolonic instillation sensitizes the intestinal mucosa, leading to increase epithelial permeability, haptenization of host proteins, microbial penetration, and infiltration of neutrophils, macrophages, and Th1 lymphocytes into the damaged mucosa [37]. Visceral hypersensitivity caused by the DNBS administration and subsequent intestinal damage is long lasting [37,38], and persists even after the resolution of the inflammatory acute phase [36,39,40]. For this reason, this animal model is frequently used as a postinflammatory IBS/IBDs model [39,41,42].

Abdominal hypersensitivity related to these pathologies is currently treated by different, often unsatisfactory, approaches. In IBD patients, pain is managed by nonsteroidal anti-inflammatory drugs, opioids, antispasmodics, anticonvulsants, tricyclic antidepressants, or by a generic intensification of disease therapy (aminosalicylates, corticosteroids, immunomodulators, biologic agents, antibiotics, and probiotics [43].

IBS-related visceral pain treatment is approached though dietary measures (fiber supplementation, low fermentable oligosaccharides, disaccharides, monosaccharides, and polyols diet) and pharmacological resources, such as antispasmodics, antidepressants (TCA and SSRI), 5-HT3 antagonists (alosetron), nonabsorbed antibiotic (rifaximin), secretagogues (lubiprostone, linaclotide), μ and κ agonist, δ antagonist, H1 antagonist (ebastine), GABAergic agents (gabapentin and pregabalin), and peppermint oil [24].

Most of these therapies cannot be considered for the long-term management of visceral pain because they offer, in many patients, little benefit joined to significant side effects [44,45,46]. For these reasons, a growing amount of evidence confirms that substances of natural origin can exert potent protective benefits, with fewer undesirable effects in conditions of acute or chronic intestinal inflammation [47]. It was demonstrated that a diet rich in fruits and vegetables is able to reduce the incidence and prevalence of IBDs [48,49], since, in addition to affecting host immunity and intestinal barrier function, dietary nutrients can modify the composition and function of the gut microbiota [50]. Recently, we reported the visceral pain-relieving effect of a system of molecules of vegetal origin [36].

Since ancient times, due to its reported benefits to human health, the pomegranate has drawn great interest from the consumers and researchers [51]. Nowadays, the pomegranate is used for functional food ingredients and dietary supplements in various forms, and numerous phytochemicals have been identified in its different parts [52]. Pomegranate seeds, fruits, juice, and peel (that represents for half of its weight) all possess therapeutic health-promoting constituents in the form of various bioactive compounds such as ellagic acid, ellagitannins, punicalagins, punicic acid, flavonoids, anthocyanidins, anthocyanins, estrogenic flavonols, other fatty acids, and flavones suitable for remedial applications [53,54,55]. This particular phytochemical profile has been related to the wide range of biological properties of pomegranate products [56], moving pomegranate into the spotlight of nutritional and pharmacological research. Indeed, scientific evidence shows that many of its constituents exhibit antimicrobial, antidiarrheal, antioxidant, antiulcer, and anticancer properties [57,58].

Pomegranate peels used to be one of the most valuable byproducts of the food industry, and have now attracted much attention due to their wide range of bioactivities. Polysaccharides extracted from pomegranate peels are known to possess excellent antioxidant and immunomodulatory properties [59]. Joseph and colleagues [60] demonstrated that a galactomannan extracted from the peel of *Punica granatum* L. fruit presents radical scavenging activity [61] and prebiotic effects, stimulating the growth of beneficial bacteria in the colon and maintaining a good state of health of the intestine [62]. In a dextran-sulfate-induced colitis animal model, Yue and colleagues [63] showed that the polysaccharides ameliorated the inflammatory response through lowering TNF-α, IL-1β, IL-6, and MPO activity and increased AMPK activity. In 2016, Hung [64] and Segarra [65] showed that the administration of polysaccharides reduced the clinical score in different colitis animal models. Furthermore, the polysaccharides were able to prevent intestinal mucosal damage through Epidermal Growth Factor modulation, with a significant enhancement of mucus synthesis [66,67].

Another class of pomegranate compounds that has attracted much attention, are polyphenols [68]; they can be subdivided into flavonoids and nonflavonoids, but are mainly constituted by ellagitannins, particularly punicalgins. Polyphenols can directly act as antioxidants prior to absorption in the gut lumen (where their concentration is high), removing reactive oxygen species, or following absorption, via their influence on nuclear receptors and gene expression [69]. In addition, polyphenols strengthen intestinal barrier properties via fostering the growth of health beneficial bacteria and influence directly the permeability of the mucosa, via acting on the tight junctions [70,71,72,73]. Overall, polyphenols can improve gene expression related to the production of proteins required for tight junction integrity, including claudin-5, occludin, and zona occludens-1 [49].

Hydrolyzable tannins are among the most studied phytochemicals in pomegranate; they can be further grouped into ellagitannins and gallotannins [52]. Overall, more than 60 hydrolyzable tannins have been identified from pomegranate, and its peel is particularly rich in ellagitannins [74]. Cerdá and colleagues [75] showed that the administration of ellagitannins in rats decreased the MMP-9 level, with the prevention of NFκB promoter activity. In addition, ellagitannins inhibit the activation of inflammatory pathways such as MAPK [76]. They can also inhibit angiogenesis through the downregulation of vascular endothelial growth factor in cancers [54].

Ellagitannins are not absorbed as such, but they are hydrolyzed in ellagic acid at the intestinal level; ellagic acid and punicalagin are also poorly absorbed in the stomach and small intestine, but they are further metabolized by the gut microbiota to urilithins, mainly Uro-A and Uro-B [77,78,79,80]. Emerging experimental evidence has suggested that urolithins play a major role in the anticancer, anti-inflammatory, and antiaging activities of pomegranate fruit and products [81,82]. Recently, Singh and colleagues [82] showed that urolithin A can mitigate IBD by increasing the proteins that tighten epithelial cell junctions in the gut by reducing intestine inflammation.

In our DNBS-induced colitis model, the higher potency of ellagitannins in comparison to polysaccharide fractions was highlighted; in fact, the effect of ellagitannins was comparable to that of the total decoction, while polysaccharides needed a dose nine times higher than that present in the decoction to reach the same efficacy. Nevertheless, it is important to note that all three products were active and able to protect the intestinal mucosa, reducing the macroscopic alterations, irritation, and the inflammatory infiltrate in the tissue. Furthermore, they reduced fibrosis state (a serious clinical complication affecting many IBDs patients), as well as the overall amount of mast cells and the number of degranulated mast cells in the mucosal stroma. Mast cell hyperplasia and activation lead to abnormal gastrointestinal sensitivity, motility, and secretion, which in turn contribute to the hallmark symptoms of IBDs (abdominal pain and/or discomfort, bloating, and abnormal bowel function). Furthermore, Krammer and colleagues support the idea that mast cells are also involved in IBS pathophysiology as key players in the interplay between psychological factors and the frequency and severity of IBS symptoms [83].

In conclusion, we show that ellagitannins of pomegranate mesocarp play a main role for most of the beneficial effects of pomegranate on DNBS-induced colitis, and the polysaccharide fraction contributes and supports this activity.

More studies are needed to clarify the specific role of these bioactive fractions as well as the mechanisms underlying the relieving effects described here. Nevertheless, on the basis of the present results and supported by literature evidence, we conclude that pomegranate mesocarp preparations could represent a nutraceutical approach, alone or to complement conventional therapies, for promoting abdominal pain relief in gut-related pathologies.

## 4. Materials and Methods

### 4.1. Plant Material

A batch of 20 kg of fresh fruits of the Wonderful variety was harvested at full ripening in the Apulian region, and was used to prepare the decoction. The different tissues of the fruits were separated; the exocarp was removed from the peel to obtain the mesocarp alone. According to Khatib et al. [84], the fresh mesocarp (1 kg) was boiled in 8 L of ultrapure water, for 60 min. The supernatant was recovered after cooling and centrifugation (4500 rpm, 10 min at 4 °C). A part of the clear solution was freeze dried to obtain the dry decoction of mesocarp and the sample whole decoction. An aliquot of the supernatant was added with three volumes of ethanol, and kept at 0 °C for 60 min to induce the precipitation of the polysaccharides, then recovered after centrifugation (4500 rpm, 8 min, 4 °C); the precipitate was freeze-dried to obtain the polysaccharides sample. The water–ethanol solution remaining after polysaccharide removal was concentrated by rotavapor to eliminate the ethanol, resulting in a liquid sample containing the ellagitannins.

The ellagitannins in dry decoction and polysaccharides were determined using a HP 1200L liquid chromatograph equipped with a DAD detector (Agilent Technologies, Palo Alto, CA, USA). A Kinetex C18 column (30 × 3 mm, 2.6 µm, Agilent-USA) was used, applying a linear multistep gradient. Solvent A from 5% to 25% in 8 min, then 10 min at A 25%, in 2 min reached 95%, and held for 6 min. Equilibration time was 10 min; the total time of analysis was 28 min, solvent A was CH_3_CN and solvent B was H_2_O acidified by HCOOH (3% *v*/*v*), the injection volume was 2 μL. The ellagitannins were quantified according to their maximum of absorption at either 380 nm using a five-point calibration curve of a racemic mixture of α and β-punicalagins (purity ≥ 99%, linearity range 2–5 µg, R2 = 1.000), and at 370 nm using a five-point calibration curve of ellagic acid (purity 95%) (linearity range 0–1.7 µg, R2 = 1.000).

### 4.2. Selection of the Tested Doses

Three samples were used for the animal treatment, and the daily dose of each sample was determined according to the composition of the whole decoction. The yields in dry decoction on dry mesocarp were approximatively 70%; the dry polysaccharides were 11.5% on dry decoction. The total ellagitannins in the decoction were 15.1%; according to the HPLC data, each mL of the ET sample contained 320 mg ellagitannins. The daily dose for the sample containing the ellagitannins was chosen in order to test the same amount of the ellagitannins in the whole decoction: 300 mg of whole decoction was equivalent to 45.3 mg ellagitannins, corresponding to 0.94 mL of ellagitannins sample. Regarding the polysaccharides sample and taking into account the scant data in the literature on the bioactivities of the polysaccharides of pomegranate, the chosen dose was 300 mg kg^−1^; this dose was almost nine times higher with respect to the polysaccharide amount in 300 mg of the whole dry decoction.

### 4.3. Animals

Male Sprague–Dawley rats (Envigo, Varese, Italy) weighing approximately 220–250 g at the beginning of the experimental procedure were used. Animals were housed in CeSAL (Centro Stabulazione Animali da Laboratorio, University of Florence) and used at least one week after their arrival. Four rats were housed per cage (size 26 × 41 cm); animals were fed a standard laboratory diet (24% protein, 58% carbohydrate, 18% fat; Envigo, Varese, Italy) and tap water ad libitum, and kept at 23 ± 1 °C with a 12 h light/dark cycle, starting at 7 a.m. All animal manipulations were carried out according to the Directive 2010/63/EU of the European parliament and of the European Union council (22 September 2010) on the protection of animals used for scientific purposes. The ethical policy of the University of Florence complies with the Guide for the Care and Use of Laboratory Animals of the US National Institutes of Health (NIH Publication No. 85-23, revised 1996; University of Florence assurance number: A5278-01). Formal approval to conduct the described experiments was obtained from Ministry of Health (approval code: 543/2017, approved on 3 July 2017) and from the Animal Subjects Review Board of the University of Florence. Experiments involving animals have been reported according to ARRIVE guidelines [85]. All efforts were made to minimize animal suffering and to reduce the number of animals used.

### 4.4. Induction of Colitis

Colitis was induced in accordance with the method described previously by Fornai et al., [86] with minor changes. In brief, during a short anesthesia with isoflurane (2%), 30 mg of 2,4-dinitrobenzenesulfonic acid (DNBS; Sigma-Aldrich, Milan, Italy) dissolved in 0.25 mL of 50% ethanol was intrarectally administered via a polyethylene PE-60 catheter inserted 8 cm proximal to the anus. Control rats received 0.25 mL of saline solution.

### 4.5. Drug Administrations

The pomegranate whole decoction (300 mg kg^−1^), polysaccharides (300 mg kg^−1^), and ellagitannins (45 mg kg^−1^) fractions were orally administered once daily for 14 days, starting from DNBS injection. The compounds were suspended in 1% carboxymethylcellulose sodium salt (CMC) for oral administrations. Control rats were daily treated with 1% CMC.

### 4.6. Assessment of Visceral Sensitivity by Visceromotor Response (VMR)

The Visceromotor Response (VMR) to colorectal balloon distension (CRD) was used as an objective measure of visceral sensitivity in animals. Two EMG electrodes were sutured into the external oblique abdominal muscle under deep anesthesia and exteriorized dorsally [87]. VMR assessment were carried out under light anesthesia (Isoflurane 2%). A lubricated latex balloon (length: 4.5 cm), assembled to an embolectomy catheter and connected to a syringe filled with water was used to perform colorectal distension. A syringe was used to fill the balloon placed into the colon with various volumes of water (0.5, 1, 2, 3 mL). The electrodes were relayed to a data acquisition system and the corresponding EMG signal was recorded, amplified, and filtered (Animal Bio Amp, ADInstruments, Colorado Springs, CO, USA), digitized (PowerLab 4/35, ADIinstruments, Colorado Springs, CO, USA), analyzed, and quantified using LabChart 8 (ADInstruments, Colorado Springs, CO, USA). To quantify the magnitude of the VMR at each distension volume, the area under the curve (AUC) immediately before the distension (30 s) was subtracted from the AUC during the balloon distension (30 s), and responses were expressed as percentage increase from the baseline. The time elapsed between two consecutive distensions was 5 min. The measurements were carried out 7 and 14 days after DNBS administration.

### 4.7. Assessment of Visceral Sensitivity by Abdominal Withdrawal Reflex (AWR)

Behavioral responses to CRD were assessed via Abdominal Withdrawal Reflex (AWR) measurement using a semiquantitative score in conscious animals [88]. Briefly, rats were anesthetized with isoflurane, and a lubricated latex balloon (length: 4.5 cm), attached to polyethylene tubing, assembled to an embolectomy catheter and connected to a syringe filled with water was inserted through the anus into the rectum and descending colon of adult rats. The tubing was taped to the tail to hold the balloon in place. Then, rats were allowed to recover from the anesthesia for 30 min. AWR measurement consisted of visual observation of animal responses to graded CRD (0.5, 1, 2, 3 mL) blinded observers who assigned an AWR score: no behavioral response to colorectal distention (0); immobile during colorectal distention and occasional head clinching at stimulus onset (1); mild contraction of the abdominal muscles but absence of abdomen lifting from the platform (2); observed strong contraction of the abdominal muscles and lifting of the abdomen off the platform (3); arching of the body and lifting of the pelvic structures and scrotum (4). The measurements were carried out 14 days after DNBS administration.

### 4.8. Macroscopic and Microscopic Analysis of Tissue Damage

On day 14, the animals were sacrificed, the colorectal portion of the intestine was removed and processed for both macroscopic and microscopic analyses, in accordance with the criteria previously reported by Antonioli et al. [89]. The macroscopic criteria were: presence of adhesions between colon and other intra-abdominal organs (0–2); consistency of colonic fecal material (indirect marker of diarrhea; 0–2); thickening of colonic wall (mm); presence and extension of hyperemia and macroscopic mucosal damage (0–5).

The intestine samples were then fixed in formalin at 4% for 24 h, dehydrated in alcohol, included in paraffin, and finally cut into 5 μm sections. Microscopic evaluations were performed on sections stained with: hematoxylin and eosin, for conventional histopathological analysis; picrosirius red (PR) staining for collagen fibers to assess the degree of fibrosis of the colonic mucosa; tryptase immunohistochemistry, to evaluate the extent of granule release by mast cells in the colonic mucosa. Micrographs to be analyzed were taken using Nikon Olympus BX40 and a 400× objective equipped with NIS F3.00 Imaging Software^®^. For PR, a morphometric quantitative evaluation of the staining intensity was performed on the digital images using the free-share ImageJ 1.42 image analysis software (http://rsb.info.nih.gov/ij). Volume density (VD) measurements of the details of interest were carried out upon selection of an appropriate threshold to include the stained surface area. The reported values are the means ± SEM of the measurements of individual animals (at least five images each) from the different experimental groups.

### 4.9. Statistical Analysis

Behavioral measurements were performed on six rats for each treatment carried out in two different experimental sets. All assessments were made by researchers blinded to the animals’ treatments. Results were expressed as means ± S.E.M. and the analysis of variance was performed by one-way ANOVA. A Bonferroni’s significant difference procedure was used as post-hoc comparison. *P* values of less than 0.05 or 0.01 were considered significant. Data were analyzed using the “Origin 9” software (OriginLab, Northampton, MA, USA).

## Abbreviations

AWR	Abdominal Withdrawal Reflex
CRD	Colon Rectal Distension
DNBS	2,4-dinitrobenzenesulfonic acid
IBDs	Inflammatory Bowel Diseases
IBS	Irritable Bowel Syndrome
VMR	Visceromotor Response
